# Nanocomposite Coatings for Anti-Corrosion Properties of Metallic Substrates

**DOI:** 10.3390/ma16145092

**Published:** 2023-07-19

**Authors:** Liana Maria Muresan

**Affiliations:** Department of Chemical Engineering, Faculty of Chemistry and Chemical Engineering, “Babes-Bolyai” University, 400028 Cluj-Napoca, Romania; liana.muresan@ubbcluj.ro

**Keywords:** nanocomposite coatings, metallic substrates, reinforcement materials, anti-corrosion properties

## Abstract

Nanocomposites are high-performance materials with exceptional characteristics that possess properties that their individual constituents, by themselves, cannot provide. They have useful applications in many fields, ranging from membrane processes to fuel cells, biomedical devices, and anti-corrosion protection. Well-tailored nanocomposites are promising materials for anti-corrosion coatings on metals and alloys, exhibiting simple barrier protection or even smart auto-responsive and self-healing functionalities. Nanocomposite coatings can be prepared by using a large variety of matrices and reinforcement materials, often acting in synergy. In this context, recent advances in the preparation and characterization of corrosion-resistant nanocomposite coatings based on metallic, polymeric, and ceramic matrices, as well as the incorporation of various reinforcement materials, are reviewed. The review presents the most important materials used as matrices for nanocomposites (metals, polymers, and ceramics), the most popular fillers (nanoparticles, nanotubes, nanowires, nanorods, nanoplatelets, nanosheets, nanofilms, or nanocapsules), and their combinations. Some of the most important characteristics and applications of nanocomposite coatings, as well as the challenges for future research, are briefly discussed.

## 1. Introduction

Corrosion is a natural process causing degradation, failure, and hazards in many industrial processes and domestic systems. It cannot be completely avoided, but intense research is carried out to minimize its effects. Among other methods, the coating of metallic substrates is most widely used for preventing, hindering, or controlling corrosion due to a large variety of coating materials and coating methods, which can be used in specific applications. In this regard, nanocomposite coatings represent a modern and performing category of materials with outstanding properties that improve significantly the mechanical, tribological, and anti-corrosion properties of the metallic substrates.

Nanocomposite coatings consist of at least two immiscible phases (at least one being nanosized) separated by an interface region. The main component in these coatings is the matrix, in which a nanofiller or a reinforcement material is dispersed in an attempt to obtain a final product with superior properties compared to each component material individually. The main qualities of nanocomposites are strength, light weight, corrosion resistance, design flexibility, and durability. Nanostructured coatings exhibit different properties from the conventional coatings with larger grains, which enabled them to be superior to their counterparts in what the mechanical and corrosion properties are concerned [1]. Due to the small dimensions of the nanoparticles acting as reinforcement materials, they fill in the spaces, block the corrosive species from diffusing towards the surface of the substrate, and offer better protection against corrosion. Moreover, strong interactions can occur between the filler and the host matrix, which result in lower porosity and lower cracking potential of the nanocomposite coatings. In the case of polymeric coatings, nanoparticles can prevent disaggregation of the polymer during curing and reduce the trend for the coating to blister or delaminate [2]. Thanks to nanotechnologies, nanocomposite coatings with different functionalities ranging from the simple barrier protection property to smart auto-responsive and self-healing functionalities are one of the rapidly developing areas with many applications in industrial and domestic fields. The aim of the review is to present the most important materials used as matrices for nanocomposites and the most popular fillers, as well as their combinations. Some of the most important characteristics and applications of nanocomposites used as anti-corrosion coatings are briefly discussed in what follows.

## 2. Matrices

The most important component of a composite coating is the matrix, which can be metallic, polymeric, or ceramic. The matrix binds the reinforcement material, transfers loads between the components, provides the composite’s net shape, and determines its surface quality. There is a large variety of materials that can be used as matrices, depending on the destination of the composite and on the experimental conditions in which the material is used. Metals, polymers, or ceramics compete for anti-corrosion coatings on metals and are used depending on the application area of the system and the exploiting conditions.

### 2.1. Metals

Nanocrystalline metal matrices had received special attention due to their ability to act as hosts for various nanofillers, which significantly improves their corrosion resistance. Corrosion protection with nanocomposite metallic coatings is achieved by building a compact barrier to prevent charge transfers, which minimizes the permeability of oxygen and ion transportation. The most common metals used as matrices in nanostructured composite coatings are zinc, nickel, copper, and alloys such as Zn–Ni, Ni–Co, etc. Steel [3,4,5], aluminum [6,7,8], and magnesium [9] are the most frequently used substrates to be protected with metal matrix composites. It is common knowledge that zinc acts as sacrificial coatings for ferrous substrates, and that zinc composites provide even superior mechanical properties and better sacrificial protection to steel than pure zinc since they corrode slower. Moreover, they exhibit excellent mechanical and tribological properties and paint ability. Nickel is also used as a matrix for composite coatings on steel because of its enhanced hardness, wear resistance, self-lubrication, and corrosion resistance. However, the number of metals used as matrices is much larger.

Various metallic composites are prepared starting from the above-mentioned matrices due to their superior properties as compared to the native metals. Most of the metallic coatings are deposited on the substrates by electroplating, which is one of the most efficient methods to form metallic layers due to its simplicity and process stability, widely used in industries, but other methods are also used.

### 2.2. Polymers

Polymer-based coatings are best able to respond to obstructive environmental rules, and their use entails no threat for the consumer. Polymer materials are widely used in industry due to their ease of production, low cost, small specific weight, and often ductile nature. However, they have some disadvantages when compared to metals and ceramics, such as lower modulus and strength [10].

The most common way for polymeric coatings preparation on metals are the sol–gel method [11,12], plasma-based coating methods [13], electrodeposition (in the case of conducting polymers such as polyaniline, polypyrole etc.) [14], layer by layer deposition (LBL) [15], spray coating [13], etc.

A wide range of polymers is used for the anticorrosion protection of metals and can act as matrices for composite materials. Among them, one can count biopolymers (e.g., chitosan, sodium alginate, etc.) and a large number of synthetic polymers (e.g., epoxy, polyaniline, polypyrole, etc.). Only some of them were selected to be shortly presented below.

#### 2.2.1. Biopolymers

Some naturally occurring organic polymers, such as lignin, tannin, inulin, extracellular polymeric substance, cellulose, carrageenan, chitosan, gum and polydopamine, have been reported as corrosion inhibitors for steel and other metals, such as zinc or copper [16,17,18]. They act as a barrier against corrosion due to the presence of heteroatoms such as oxygen, nitrogen, or sulfur in a conjugated ring system, which enables the adsorption of the organic molecule on the metal surface [19]. Biopolymer coatings can be easily functionalized and exhibit outstanding potential in various biomedical applications. They present numerous advantages, as they are biodegradable, bioactive, and nontoxic; they also possess good sorption and adhesion properties. They may facilitate cell production, tissue development, repair, and delivery of biomolecules, such as antimicrobial agents, active molecules, growth factors, and drugs [20], which are important properties in the case of medical implants. Biopolymers enhance corrosion and wear resistance of the substrate. In some cases, they can act as switchable smart materials serving as biomimetic surfaces in the human body. Some biopolymers can be used to blend with water-borne polymers to improve the thermal, mechanical, and protection properties of the host polymer [21].

One of the mostly used biopolymers in corrosion inhibition of mild steel, copper, and zinc without causing environmental problems is chitosan. Chitosan is a natural linear polysaccharide composed of β-linked D-glucosamine and N-acetyl-D-glucosamine units, which are obtained from the outer skeleton of crustaceans processed in alkaline media, for example, in sodium hydroxide. Chitosan is biocompatible, has good chemical resistance, antimicrobial properties, and mechanical strength, and it is thermally stable [22]. Chitosan coatings exert corrosion protection by acting as a physical barrier that retards diffusion of aggressive elements through the coating. Moreover, they inhibit the charge transfer between local anodic and cathodic sites of the surface [23]. Chitosan can be crosslinked with anionic [24] or covalent [25] species and can be loaded with corrosion inhibitors that can be released and heal defects occurring in the coating. Some researchers enhance the inhibition action of chitosan with composite formation. For example, the efficiency of chitosan and boron nitride combination in corrosion protection of mild steel in acidic media was recently reported [26].

A combination of bioactive materials with antibiotics can be efficient in the case of biomedical applications such as the handling of osteomyelitis. This combination plays a dual role as local drug delivery and as bone cell growth systems. As an example, chitosan-lysine biopolymers loaded with gentamycin and possessing enhanced bioactive and corrosion-resistance properties were coated on Ti for orthopedic implants [27].

Another biopolymer used as matrix for corrosion-resistant composites is sodium alginate. Sodium alginate is an anionic polysaccharide extracted from the cell walls of seaweed. The sodium alginate exhibits properties like water solubility, biocompatibility, biodegradability, and non-toxicity. The presence of carboxylate functional group in the alginate moiety favors oxygen linkage with metal cations in solution [28]. Sodium alginate was reported as a promising biopolymer for corrosion protection of carbon steel in saline medium [19] and of titanium, in combination with chitosan [29]. The presence of adsorbed alginate and albumin on aluminum coatings inhibits adhesion of Escherichia coli and improves the anti-corrosion resistance of the coatings through modifying the superficial properties of the coatings, such as hydrophilicity/hydrophobicity [30].

#### 2.2.2. Synthetic Polymers

Synthetic polymers, such as epoxy materials [31], polystyrene [32], polyaniline (PANI) [33,34], polypyrrole (pPy) [35,36], and polyesters [37], are successful candidates for corrosion-resistant coatings due to their excellent scratch hardness, good adhesion, and strength and wear endurance. Consequently, they are attractive matrices for composites hosting various reinforcement materials.

Electrically conductive polymers (such as polyaniline, polythiophene, and poypyrrole) can isolate the metal surface from the surrounding environment providing barrier protection or can build a passive layer of metal oxide on the substrate providing anodic protection [35,38]. They can be easily deposited on a metallic substrate by electrodeposition/electropolymerization.

Self-healing polymer matrices were also developed in an attempt to prolong the lifetime of the protective coatings by repairing structural defects upon damage. Intrinsic self-healing polymers possess chain mobility and entanglement, or can suffer reversible polymerizations, melting of thermoplastic phases, hydrogen bonding, or ionic interactions to initiate the process [39]. Some examples are urea polymer networks [40], polyurethanes [41], and epoxy-based materials [42].

It is also worth mentioning another category of polymers used as corrosion-resistant coatings, the so-called water-borne polymer coatings. They use water as a solvent to disperse resin and have valuable properties, including low-toxicity and viscosity, easy cleaning, and environmental friendliness (e.g., water-based alkyds coatings) [1]. In addition to their low toxicity, water-borne coatings are less inflammable due to their high-water content, and they reduce or completely eliminate hazardous waste disposal. Water-borne coatings are, in present, comparable to solvent-borne coatings due to the latest progresses made on their chemistry.

More than 80% of the market for water-borne coatings is occupied by acrylic coatings. They could be combined with different fillers to give resistant composite coatings for metals, especially for steel. For example, water-borne acrylic paint system hosting nanoceria nanoparticles was successfully used for corrosion protection of steel [43].

Water-borne epoxy coatings were also developed. For example, epoxy coatings containing a novel mussel-inspired adhesive polymer [44] and water-borne epoxy/polyacrylate composites [45] were proven to be promising candidates as matrices of anticorrosion coatings on steel.

Polymeric coatings can be applied on metallic substrates by brush painting, dip-coating, spraying, etc.

### 2.3. Ceramics

Another class of materials frequently used as matrices for nanocomposite coatings is ceramics. Ceramics have been employed in many industrial fields due to their excellent temperature stability, low density, high hardness, good corrosion, and wear resistance in demanding thermal and mechanical requirements. Like other corrosion-resistant coatings, the ceramic coatings isolate the corrosion solution from the substrate, providing an effective physical barrier on the surface of metallic materials. While choosing the material for ceramic-resistant coatings, an important criterion is the thermal expansion coefficient (TEC). Thus, to avoid cracks and obtain the desired properties, the optimal ceramic coating should have a TEC close to that of the substrate [46].

Ceramic matrices exist as oxide and non-oxide types. As oxide components, different compounds such as Al_2_O_3_ or TiO_2_, or combinations such as Al_2_O_3_–SiO_2_ and Al_2_O_3_–ZrO_2_ are commonly used. They exhibit lower maximum application temperatures than non-oxide type ceramics but are easier to fabricate, less expensive, and resistant to oxidation [47].

TiO_2_ coating is considered to be an excellent oxidic anti-corrosion protective layer for Ti-based substrates. It deserves special mention due to its exceptional photoactive antibacterial property and hemocompatibility, which make it useful in biomedical implants [48,49]. TiO_2_ can be directly formed on the surface of Ti and Ti alloys by electrochemical anodization, spark anodization, or anodization of Ti metal in a HF-containing electrolyte when self-organized TiO_2_ nanotubes are formed [50]. Through anodic oxidation of Ti, various morphologies such as meso-sponge and nanotube layers can be produced [49]. Morphology of the coatings is important and can be controlled by monitoring the experimental parameters during the coating preparation. For example, mesoporous layers of titania prepared by anodic oxidation should be etched to provide open channels and annealed to favor hydroxyapatite formation, which is an indicator of bioactivity of the surface during bone growth.

Non-oxide ceramics are covalent-bonded ceramics with low values of thermal expansion and high thermal conductivity. They include carbides, nitrides, and borides like SiC [51], Si_3_N_4_, [52], TiB_2_ [53], etc. Hydroxyapatite (HA) is a non-oxide ceramic material, which is bioactive and commonly used in bone tissue engineering. It was reported that 316 L of stainless steel is well-protected by HA coating against corrosion, and at the same time, the bone osseointegration and biocompatibility of the metallic implant are improved [54].

Ceramic coatings can be directly generated on the surface of aluminum, magnesium, and titanium by plasma electrolytic oxidation (PEO) [55,56,57]. PEO is a complex process in which electrochemical oxidation is performed with oxide film formation, dissolution, and dielectric breakdown. The properties of coatings produced by PEO mainly depend on the type of power source, the applied current density, the compositions, the concentration of the electrolyte, and the nature of the metallic substrate [58]. Other obtaining methods are sol–gel coating [59], electrophoretic deposition [60], thermokinetic deposition processes [61], etc.

## 3. Reinforcement Materials/Fillers

The role of the reinforcement in a composite material is mainly one of increasing the mechanical and anti-corrosion properties of the system, but the intrinsic properties of nanofillers, as well as their size, morphology, chemical functional groups, and their amounts, influence significantly many more properties of nanocomposites. The dispersion of nanofillers in a metallic, ceramic, or polymeric matrix provides coatings with improved characteristics such as hardness, corrosion, and wear resistance, as well as improved thermal stability. Optimum concentration of filler material well-dispersed in the metal matrix can extend the penetration path of the corrosive ions (generating a high tortuosity factor) and prolongs the lifetime of metals [5]. Moreover, the nanoparticles incorporated into polymers showed an increase of the integrity and lifetime of coatings thanks to the filling up of cracks and microcavities in the coatings. However, uniform dispersion is a difficult task due to the agglomeration tendency of the nanomaterials.

**Figure 1:**
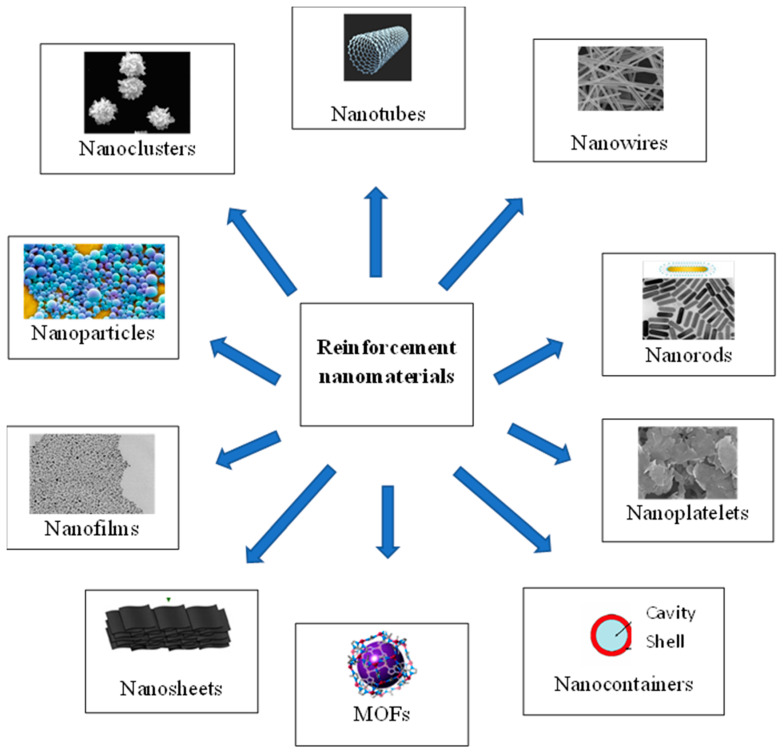
Main types of nano-sized reinforcement materials used for nanocomposite coatings.

Reinforcement materials mostly used are in the form of nanoparticles, nanotubes, nanowires, nanorods, nanoplatelets, nanosheets, nanofilms, or nanocapsules [1]. The most important types of reinforcing materials are summarized in Figure 1, and the most frequently used will be briefly discussed in the next sections.

### 3.1. Nanoparticles (NPs)

Nanoparticles are materials that have size ranges from 1 to 100 nm. NPs of different families, shapes, dimensions, and surface functional groups have been used in developing advanced composite coatings after incorporation in different matrices.

The main difficulties encountered in obtaining nanocomposite coatings with high-protection efficiency incorporating nanoparticles are, generally, the low degree of particles incorporation in the matrix, the agglomeration of the particles and, hence, the effort to ensure a uniform distribution of the fillers in the coating. The smaller the particle size, the higher the agglomeration tendency and difficulty to obtain uniform deposits. Some of these problems can be, at least partially, solved by modifying the surface properties of the particles. Nowadays, various methods have been developed to ensure the stability of the particles in the matrix, such as the use of surface-active agents, surface modifiers, capping agents, dopants etc.

The nanoparticles can be amorphous [62] or crystalline [63] with various geometries: spherical [64], cubic [65], tubular [66], etc. Besides conventional nanomaterials, such as oxides [67,68,69,70], or metals [71,72], new native or functionalized nanoparticles such as carbon nanotubes [73], graphene [74], graphene oxide [75], or organo-grafted nanoparticles [76,77] represent a new generation of nanomaterials used as fillers in composite coatings. 

Graphene is a 2D carbon nanomaterial that possesses unique electrical, optical, and mechanical properties but a low dispersibility in matrices due to the lack of surface functional groups. On the contrary, graphene oxide (GO) preserves the exceptional properties of graphene, having additionally abundant surface functional groups and, consequently, good dispersibility and solubility in solvents, which makes it promising nano-scale filler for a next generation of functional composite materials. The functionalization of graphene oxide with a high surface area is found to improve the dispersion degree and, hence, enhances stability and mechanical properties of the coatings [5]. The fine nanoparticles dispersed in coatings can fill in cavities, increase the cross-linking density, and prevent matrix disaggregation during curing, offering solutions to enhancing the integrity and durability of coatings [78].

Besides graphene, other popular oxide nanoparticles used as fillers in composite coatings are TiO_2_ [69], ZnO [79,80], SiO_2_ [8], Al_2_O_3_ [81], ZrO_2_ [68], CeO_2_/ZrO_2_ [67], and TiO_2_·CeO_2_ binary oxides [82]. The grafting of organic molecules on oxide nanoparticles (e.g., PANI on Al_2_O_3_ [76]) or ferocene onto CeO_2_ [77]) and/or doping with inhibitors (e.g., Ce nitrate doped alumina/polyaniline nanoparticles into epoxy coating [76]) help the uniform dispersion and incorporation of NPs in the matrix, providing active protection against corrosion. Recently, core-shell nanosized architectures have been synthesized [83], offering the possibility to place corrosion inhibitors in the core of the system for a controlled release and develop the self-healing concept [84].

A short selection of the reported nanoparticles used in composite corrosion resistant coatings is presented in Table 1.

It can be observed that the NP concentration used to prepare the nanocomposites is variable and depends on the matrix and of the filler’s nature and on the preparation method.

### 3.2. Nanotubes

A nanotube is a nanometer-scale hollow tube-like structure made of different materials, such as carbon, titania, boron nitride, silicon, silicon carbide, etc. Most research has been focused on carbon nanotubes (CNTs), which exhibit exceptional electrical and thermal conductivity, and exceptional tensile strength and versatility. In addition, they are easily chemically modified and functionalized. CNTs can be single walled (SWCNTs), with a diameter < 1 nm, and multi-walled (MWCNTs), consisting of concentrically interlinked nanotubes with diameters > 100 nm and length exceeding their diameter (μm, or even mm). Just like graphite, carbon nanotubes resist any chemical attack, except if they are simultaneously exposed to oxygen and high temperatures. This property makes them enormously resistant to corrosion. Therefore, they can function as anti-corrosion filler, making them successful candidates as reinforcements in composite corrosion resistant coatings after embedding them in various matrices.

CNTs can fill the holes of metal- and polymer-matrix composites by forming a passive layer on metals and promoting sacrificial protection in zinc-rich polymer (ZRP) coatings [92]. MWCNTs improve the mechanical strength, decrease the porosity of epoxy resin matrices, and increase the adhesion of the coating [93].

The functionalization of CNT surfaces brings great improvement in the CNT properties by decreasing their agglomeration tendency, increasing the interactions with solvent molecules, and, hence, favoring their dispersion in a polymeric matrix [94]. Functionalization of CNTs with ester-containing surfactants led to better anti-corrosion protection of mild steel as a result of further dispersing ability [95]. CNTs doping with other materials such as polydopamine, [96], organic phosphoric acid [97], or rare-earth salts [98] can confer excellent properties and stability to the resulting composites. Moreover, the CNT-doped composites showed promising fatigue resistance and increased adhesion between the coatings and metals.

TiO_2_ nanotubes also deserve a special mention due to their use in the fabrication of quality biomedical implants. Titania mineralogical types of anatase and rutile are successful materials for the fabrication of resistant coatings on Ti and Ti alloys substrates due to their thermodynamic stability, chemical inertia, and low solubility in body fluids [99]. Various films containing TiO_2_ nanostructures (nanotubes, nanosheets, nanorods, etc.) are highly hydrophilic, which leads to augmented bioactivity and an improved osseointegration behavior of materials generally used for implants. Where the bone-bonding abilities are concerned, crystalline TiO_2_ forms overcome the amorphous ones, and nanostructured layers are superior to micro-structured ones. They are obtained on the top of tinny, naturally existing TiO_2_ on the Ti surface by various methods such as electrolytic deposition, anodic oxidation, sol–gel technique, etc. Different composite layers, including TiO_2_ nanotubes with good physical and chemical properties and improved surface bioactivity prepared on the surface of Ti-based biomaterials, were also reported [100].

### 3.3. Nanocontainers

Materials with hollow, porous, or layered structures and their assemblies are often preferred as nanocontainers to be filled with polymerizable agents or inhibitors. Nanocontainers tailored to specific actions can be incorporated in different matrices (e.g., epoxy, silica etc.), which result in nanocomposite coatings with self-healing properties, especially for corrosion protection of metallic substrates (aluminum, magnesium, steel, and their alloys). During this process, the controlled release of the healing material efficiently repairs cracks that appear in the coatings. A change in the surrounding environment (e.g., pH) can trigger the delivery of the repairing agent or inhibitor from the nanocontainers at the damaged site of the coating. [101].

The encapsulation of corrosion inhibitors into protective shells is the most frequently used technique for incorporation because it presents several advantages over the use of these inhibitors in their free molecular forms. When the core material is unstable, the shell will prevent its premature degradation/altering. The slow release of the corrosion inhibitor from the nanocontainers enables long-term delivery of corrosion inhibitors and the healing of a damaged coating [102]. Organic inhibitors containing nitrogen (e.g., azole groups, amines and amino acids) are preferred [103], but natural compounds such as different plant extracts were also encapsulated in polymeric shells [104,105].

The capsules are prepared mostly from organic polymers, (biopolymers and synthetic polymers), mesoporous silica [106], inorganic clays, and polyelectrolyte multilayers. Microcapsules with the desired properties of thickness, morphology, and sizes could be tailored via proper monitoring of the preparation process parameters. 

The most used methods to encapsulate healing agents within nanocapsules are in-situ and interfacial polymerization, multi-stage emulsion polymerization, solvent evaporation, sol–gel, and electro-spraying [105]. The encapsulation procedure must take into consideration the chemical nature of the reactive healing agent to avoid the diffusion of the liquid compound captured inside and out of the capsule shell during storage. At the same time, the microcapsule walls must be sufficiently resistant to processing conditions during their incorporation into the matrix of the host composite [107].

In the last years, multicore microcapsules were prepared. These materials provide the dual action of self-healing and anticorrosion by encapsulating two corrosion inhibitors in cross-linked polymeric shells [108]. Hybrid microcontainers have also been produced (e.g., silica/polymer double-walled hybrid nanocontainers consisting of a hollow cavity, an inner wall of porous SiO_2_, and outer polymeric wall, which is stimuli-responsive) [109]. The design and preparation of polymeric, inorganic, and hybrid nanocontainers with versatile functionalities represent a challenge, providing great opportunities for the development of a new generation of stimuli responsive smart coatings with extrinsic self-healing properties.

### 3.4. Clays and Zeolites

Clay nanomaterials have received attention recently as interesting reinforcement materials to modify polymers for developing low-cost, high-performance protective coatings [110]. The swelling properties of clay minerals come from the hydration of cations in the interlayer space. Because of swelling, the clay minerals exhibit a blocking effect against the water-soluble ions entering the cavities and act as a barrier or sealing material against the surrounding environment. By incorporating clays into the coating materials, the substrate will be protected even when the coating films are damaged by cracks and pinholes [111].

Zeolites are silica–aluminate structures with relatively high chemical reactivity due to the presence of surface silanol groups. The performance of the composite films depends on the high chemical affinity of the filler toward the matrix. Zeolite fillers are usually added in a compatible matrix (e.g., silane) in order to enhance its protective action. The better corrosion resistance of silane–zeolite coating could be explained by condensation of the hydroxyl groups of zeolite surface with silane functional groups, resulting in a crosslinking of the silane network [112,113]. Thanks to their highly porous crystalline structures, zeolites can also act as nano-containers for different types of corrosion inhibitors [114,115]. In these cases, the inhibitor release during time offers a selective self-healing action in corrosion conditions [116]. Recently, halloysite nanotubes (HNTs) and modified HNTs (HNT-NH_2_ and HNT-NH_2_-PPy) were successfully introduced in an Ni–P matrix by electroless-deposition, resulting in an adherent protective coating with excellent anticorrosion properties on steel [117].

Zeolites incorporated into Mg composite scaffolds lead to higher compressive strength, corrosion resistance, and bioactivity as compared with Mg scaffolds without zeolite and could be used as a tissue engineering scaffold for possible bone regeneration applications [118]. Zinc-doped hydroxyapatite−zeolite embedded in a polymeric matrix were prepared on magnesium substrates with the aim of diminishing the corrosion rate and improving antibacterial activity [119].

### 3.5. Metal–Organic Frameworks (MOFs)

Metal–organic frameworks (MOFs) are novel organic–inorganic, highly porous structures that are composed of metal or metal-cluster-cations (so-called “nodes”) and multidentate anionic or neutral organic molecules (so-called “linkers”) [120]. MOFs possess some exceptional characteristics such as high mechanical and thermal stability, large surface area, permanent porosity, tailorable pore size and pore size distribution, chemical versatility, molecular flexibility, and ease of functionalization [121]. MOFs with 2D or 3D structures have been obtained by traditional solvothermal and non-solvothermal strategies [122]. Metallic ions as copper and manganese, which have unfilled *d* orbitals in their structures, are easy to coordinate with nitrogen atoms, so they are selected as metal ions for the synthesis of MOFs.

Since most MOF materials have high-affinity interactions with both inorganic and organic compounds, they can easily form composite anticorrosion coatings such as MOF-polymer or MOF-polymer/inorganic compound [123] to protect metal plates like Mg, Al, Zn, and their alloys.

The incorporation of MOFs into a matrix (e.g., an organic polymer) influences the properties of the coating, such as its corrosion resistance, mechanical, and dielectric properties. The presence of hydrophobic MOFs in a polymeric matrix can improve the barrier properties of the coating by hindering the access of corrosive species. MOFs are suitable hosts for corrosion inhibitors, acting as nano-reservoirs involved in self-healing processes via controlling the amount of released corrosion inhibitors.

Various molecules can be grafted on MOF surfaces in order to improve the properties of the coatings in which the MOFs are embedded. For example, water-borne epoxy resin coating with dopamine-grafted MOFs improved the toughness and strength of coating and also enhanced the adhesion force between the coating and metal substrate [33]. Composite MOF–Grafene oxide (GO) coatings can be prepared based on oxygen-containing groups (such as carboxyl, hydroxyl, epoxy, etc.) present on the GO surface, which can combine with unsaturated metal sites of MOFs to form coordination bonds [124].

## 4. Metal Matrix Nanocomposites (MMNCs)

In the last years, it has been widely accepted that the reinforcement of coatings by the addition of various particles into the metal matrix can significantly improve the coating quality. Adhesion strength and deposition efficiency are upgraded, and the porosity is reduced. Moreover, MMNCs exhibit superior corrosion resistance and are chemically stable compared to pure metallic coatings. For example, the Zn–graphene composite coating is superior to a pure Zn coating, presenting reduced grain size, less surface defects, and hillock structures, as well as a different texture [86].

Besides advantages, some drawbacks of MMNCs should be also mentioned. Thus, some of the disadvantages of MMNCs compared to monolithic metals and polymer matrix composites are a higher cost of some materials, complex fabrication methods, and immature technologies. Moreover, some practical issues have to be solved, such as compatibility between the nanoparticles and the metallic matrix, reinforcement distribution and control of interfacial properties, etc.

The methods mostly used to obtain composite metallic coatings include thermal methods (spray, internal oxidation) [125], hot dip coating [126], and electrolytic co-deposition, in a direct or pulsed current [127]. The latter is a single-step method that is superior to other methods by allowing the rigorous control of the coating thickness and of the deposition speed, the use of accessible equipment, and the work at ambient temperature [3]. Other advantages of composite electrodeposition over other coating methods are the possibility to obtain uniform deposits even on complex shapes, small quantities of waste, often encountered in dipping or spraying techniques, low levels of contamination, and the ability to produce functionally gradient material.

In spite of the numerous advantages of the electrodeposition method, several problems were identified as critical. Thus, the agglomeration of the particles in the plating bath usually determines a low degree of particle incorporation (<1%) and a non-uniform distribution of the particles in the coating. Some of these problems can be partially solved by tuning the surface properties of the particles. Additionally, organic agents can be introduced in the plating bath, and the process parameters (stirring intensity, current density, etc.) can be rigorously controlled in order to obtain high quality deposits [3].

There are numerous valuable metal/particle combinations, including metals like zinc, nickel, silver, etc., or inert reinforcement materials such as graphene, carbides, oxides, etc., which are fruitfully used in a wide variety of practical applications. Due to the great number of MMNCs reported in the literature, only a few of them are listed in Table 2, illustrating the diversity of combinations and obtaining methods.

## 5. Polymer Matrix Nanocomposites (PMNCs)

Polymer matrix nanocomposite coatings are widely used as the inclusion of nanofillers in polymeric matrices can greatly improve their corrosion resistance, thermal stability, high abrasion resistance, resistance to organic solvents, and the coating adhesion to the substrate [140]. In some cases, by the addition of nanofillers the hydrophobicity of the polymeric coating is increased, which can return an enhancement in corrosion resistance [1]. 

The polymeric matrix combined with the uniformly dispersed reinforcement materials provide a physical barrier against the attack of metallic substrates by aggressive ions present in the solution while providing a channel for the conductivity [141]. Hence, the use of PMNCs represents a simple and profitable way of improving coating properties by the addition of a small amount of suitably designed and dispersed nano-sized fillers.

Polymer nanocomposite materials have particular properties that meet special requirements. Some of the polymers can be repaired after damage by intrinsic self-healing. In this category, ionomeric co-polymers, (e.g., poly(ethylene-co-methacrylic acid copolymers with ionic segments), thermoplastic polymers (e.g., epoxy-based matrices), styrene-isoprene-styrene block copolymers [142], etc., are included. In addition, polymeric matrices are ideal hosts for dispersed catalysts and microencapsulated healing agents, which, when they react together, heal the material and avoid further crack growth by an extrinsic mechanism [143]. The essential components in such smart and self-responsive nanocomposite coatings are the inhibitors or repairing agent-loaded carriers that are released upon mechanical damage and heal the defects of the materials [38].

Different types of polymeric composites containing nanocontainers filled with inhibitors or repairing agents were reported. They include, but are not limited to, epoxy coatings embedding coconut-oil-based alkyd resin nanocontainers [105], Azadirachta indica encapsulated in urea-formaldehyde polymeric shells [104], halloysite nanocapsules modified with surfactant and filled with benzotriazole [144], etc.

To reach their maximum performance, such self-healing coatings should be prepared in optimized experimental conditions, taking into account parameters such as the concentration of inhibitors and containers, as well as the inhibition and diffusion kinetics and environmental limitations [145]. Other key factors in preparation of composite polymeric coatings are mixing during nanocomposites preparation, the presence of surfactants and their concentration, the shapes and types of nanoparticles, etc. [146]. For example, filler particles of insufficient size would lack effectiveness in providing PTFE-based coatings with wear resistance. Some polymer-nano filler combinations reported in the literature are presented in Table 3. All of the composite coatings enhance the corrosion resistance of the metallic substrates.

As can be observed from Table 3, some polymer-based coatings are hybrid (organic/inorganic) coatings. They are bicomponent [94] or multicomponent [154], aiming to bring together materials with complementary characteristics, possibly acting in synergy. This goal could also be reached by using two-layered coatings with various nanoparticles as fillers, in which the first layer is electrically conductive and the second is insulating [153].

The most common methods to prepare PMNCs are the sol–gel technique, in situ polymerization, and melt intercalation [140]. The choice of raw material and obtaining technique most suited for the processing of a particular nanocomposite depends on the application in which they are used and requires a balance between advantages and drawbacks.

## 6. Ceramic Matrix Nanocomposites (CMNCs)

Ceramic composite coatings represent an interesting alternative for metal and alloy anti-corrosion protection, especially for those working at high temperatures where some of their mechanical and tribological properties could deteriorate. Ceramic-based coatings with a thermal expansion coefficient close to that of the metallic substrate are recommended. They should be chemically inert and act effectively as a barrier between the surrounding environment and the metallic substrate by hindering corrosion and reducing significant wear issues.

Oxide systems are frequently preferred for better wear and corrosion-resistant applications because of their high-temperature resistance, insulation properties, and phase stability [46]. However, in order to diminish their brittleness and enhance the toughness, as well as for enhanced wear resistance and thermal and chemical stability of the coatings, ceramic matrix composites are preferred toward the monolithic matrix component [140]. The reinforcements used in CMNCs serve to enhance the fracture robustness of the composite material while still taking advantage of Young’s modulus and excellent strength of the ceramic matrix [155].

Ceramic nanocomposites include oxides, carbides, and nitride matrices in combination with metallic or nonmetallic elements. As already mentioned, commonly used ceramic matrices are Al_2_O_3_ [57], TiO_2_ [156], SiC [51,157], SiN [158], Si_3_N_4_ [52,159], etc., while mostly used reinforcement materials are clays [111], silica [57,160], metallic oxides [161], graphenes [159] etc., under the form of nanoparticles, whiskers, fibers, or nanotubes.

Oxide ceramic matrix composites combine high temperature stability, high strength, low density, and good corrosion resistance [47]. Oxides are often directly generated on metallic surfaces by anodic oxidation, plasma electrolytic oxidation, microarc oxidation, etc., in order to provide an appropriate environment for reinforcement material incorporation. Nanosized fillers are generally preferred due to higher surface/volume ratio and better mechanical properties of resulting nanocomposites. Thus, it was observed that the durability of nanocomposite coatings is strengthened by diminishing the particles’ size, and it reaches a maximum at a certain value of their dimensions [146].

Some examples of CMNC coatings on metals and their obtaining methods are summarized in Table 4.

Ceramic coatings containing nanocontainers filled with inhibitors or repairing agents have also been synthesized. For example, 2-mercaptobenzothiazole and 8-hydroxyquinoline corrosion inhibitors loaded in cerium–titanium oxide nanocontainers and incorporated in silica coatings significantly improved the corrosion protection properties of aluminum alloy 2024-T3 [168]. Hybrid silica layers containing embedded cyclodextrins/inhibitor nanocontainers [169] were also reported as self-healing coatings on steel.

Ceramic nanocomposites can lead to vast impact over a wide variety of fields [140], including aerospace and automotive sectors, electronic, military, and medicine, where applications of CMNCs include a new generation of medical devices based on nanocomposites with enhanced stability, hardness, strength, toughness and creep resistance, bioactive properties, and good mechanical and anti-corrosion properties.

## 7. Conclusions and Future Prospects

Nanocomposite coatings are high-performance materials that exhibit unique properties and acquire new characteristics that the individual constituents, by themselves, cannot provide. Moreover, with only a reduction in component size to nanoscale without changing the substances’ nature, materials can exhibit new properties that are impossible to obtain at a microscale or macroscale. By combining a large number of nanomaterials, it is possible to design and create new composites with improved or new physical properties.

Corrosion control by nanocomposite coatings involves preparation and use of a large variety of matrices and reinforcement materials, often acting in synergy with enhanced corrosion and wear resistance, as well as superior mechanical and tribological properties. Therefore, developing technologies at nanoscale in the future will have a great impact on our day-to-day lives.

New types of smart/stimuli-responsive/self-healing coatings are expected to provide better corrosion-protection efficiency by sensing the start of corrosion processes and replying by release of appropriate, efficient, self-healing agents. Eco-friendly, anti-corrosion coatings are foreseen to replace, at least partially, highly toxic surface treatments of materials.

Nanocomposite coatings with different functionalities, ranging from the simple barrier protection property to smart auto-responsive and self-healing functionalities, could prolong the lifetime of substrate materials and improve their characteristics.

Challenges in developing new systems to protect metallic substrates against corrosion include a better understanding of the systems’ complexity and healing mechanisms.

Further developments should be oriented towards the preparation of novel anticorrosion nanocomposites and of new healing agents, as well as the improvement of existing technologies.

## Figures and Tables

**Table 1:** Most important nanoparticles used in nanocomposite corrosion-resistant coatings.

Nanoparticles	Particles Concentration in the Composite Preparation Step	Advantages	Limitations/Drawbacks	References
Carbon nanotubes	0.4–1% CNT	- large surface area - good thermal and electrical conductivity - high mechanical resistance - versatility	- 2D material - non-uniform dispersion in the matrix	[73,85]
Graphene	100 mg L^−1^	- large specific surface area - high electrical conductivity - chemical stability	- 2D structure - difficult dispersion in aqueous solutions	[4,86]
Graphene oxide	0.1 gL^−1^ GO 0.5–5 wt.% GO	- easy dispersibility in water - easiness in manufacturing - low cost	- insulating material	[87,88]
Metals	In situ growth	- highly active surface - unique opto-electrical properties	- difficult to synthesize in pure form and with controlled size distribution - instability	[71]
Oxides	5 mM CeO_2_ 1% TiO_2_ 1, 3 and 5 g/L ZrO_2_ 0.1 g/L ZnO 1, 2, 3 wt.% ZnO 3 gL^−1^ hematite	- good mechanical abrasion, corrosion, and wear resistances - excellent mechanical strength - suitable corrosion resistance - hydrophobicity	- difficult homogeneous dispersion in coating due to agglomeration	[67,68,69,70,79,80]
Organo-grafted nanoparticles	10 gL^−1^ Fc-CeO_2_ GO-Aminothiazole (AT) and GO-2-amino-4-(1-Naphthyl)Thiazole (ANT)	- increased hydrophobicity - improved mechanical and chemical stability of the nanoparticles - enhanced corrosion inhibition properties	- delicate grafting procedure	[77,89]
Core-shell particles	3 wt.% PANI-MSNs 20 mg SiO_2_@PANI/1g silicone 5% (f-SiO_2_@RGO)	- self-healing properties - hydrophobicity - improved adhesion	- difficult synthesis	[83,90,91]

**Table 2:** Examples of MMNCs prepared with different methods on metallic substrates.

No.	Matrix	Reinforcement	Substrate	Obtaining Method	Reference
1	Zn	CeO_2_	Mild steel	Electrodeposition	[128]
2	Zn	CeO_2_	Mild steel	Hot-dip galvanization	[129]
3	Zn	TiO_2_	Steel	Electrodeposition	[130,131]
4	Zn	SiC	Steel	Electrodeposition	[132]
5	Zn	Graphene	Steel	Electrodeposition	[86]
6	Ni	Al_2_O_3_	Steel S235JR	Low-pressure cold spraying	[133]
7	Ni	Graphene	Steel	Electrodeposition	[134]
8	Ni-P	SiO_2_	Aluminum	Electrodeposition	[6]
9	Ni	WC	Low-carbon steel	Low-pressure cold spraying	[135]
10	Ni	SiO_2_	Aluminum alloy	Electrodeposition	[8]
11	Fe	WC	Low-carbon steel	Laser cladding	[136]
12	Al	SiC	ZE41 magnesium alloys	Laser cladding	[9]
13	Al	CNTs	Stainless steel	Cold-spray deposition	[137]
14	Al	Al_2_O_3_	AA 2024-T3 alloy	Low-pressure cold gas spray	[138]
15	Al	Al_2_O_3_	Al-Si Al-Si-Cu	Internal oxidation	[125]
16	Cu	Al_2_O_3_	Steel	Electrodeposition	[81]
17	Leaded brass	Al_2_O_3_	-	Stir-casting technique	[139]

**Table 3:** Representative polymer–filler combinations for anti-corrosion coatings prepared with different methods on various metallic substrates.

No.	Matrix	Reinforcement Material	Substrate	Obtaining Methods	Reference
1	Polytetrafluoroethylene	Carbon Nanotube	Stainless Steel	Doctor blade method	[147]
2	Polystyrene	Graphene	Steel	In situ miniemulsion polymerization + automatic coating	[32]
3	Epoxy	Functionalized multiwall carbon nanotubes	Carbon steel	Film applicator	[94]
4	Epoxy	Nanocellulose	Mild steel	Brushing	[148]
5	Epoxy	Graphene nanoplateles and montmorillonite	Mild steel	Doctor blade film applicator	[31]
6	Polyurethane	Multiwalled carbon nanotube (MWCNTs)	Stainless Steel	Airless spraying + curing at 70 °C	[141]
7	Chitosan	Zn	Mild Steel	Electrodeposition	[22]
8	Chitosan	Hydroxyapatite	Stainless steel	Electrophoretic Deposition	[149]
9	Chitosan	Silica + 2-mercapto-benzothiazole	Copper	Self-assembly	[150]
10	Chitosan	Montmorillonite	Mild steel	Direct melt intercalation	[151]
11	Alkyd resin	PANI -Fe_2_O_3_	Mild steel	Dip coating	[152]
12	Styrene acrylic	MWCNTs, Zn particles, graphene nanoplatelets (first layer) hexagonal boron nitrides (second layer)	Steel	Spraying	[153]
13	PANI	Fe_3_O_4_ + clay	Carbon steel	Doctor blade application	[154]

**Table 4:** Examples of corrosion-resistant CMNC coatings.

No.	Matrix	Reinforcement Material	Obtaining Method	Advantages	Reference
1	Treated high-strength aluminium alloy AA7075	α-Al_2_O_3_ and m-ZrO_2_ nanoparticles	Plasma electrolytic oxidation	Enhanced corrosion resistance	[56]
2	Zirconia toughed alumina (ZTA)	Nanosized oxide powders	High-velocity suspension flame spraying	Ease in safe nanoparticle handling	[61]
3	Nanotubular TiO_2_ layer on Ti	Hydroxiapatite (HA)	Electrodeposition	Improved stability of implant coatings	[162]
4	Al_2_O_3_/Al	SiO_2_	Plasma electrolytic oxidation of Al in dilute alkaline electrolytes with sodium silicate	Decrease of the cathodic process rate for Al corrosion reaction	[57]
5	Al_2_O_3_/Ti	Ti	Sol–gel dip coating	Improved wear resistance	[59]
6	Ceramic/metal	Ceramic particles	Electroplating	High-density and porosity-free coatings	[163]
7	[Cr+(Cr,Al)_2_O_3_] eutectic	Al-Cr_2_O_3_-Al_2_O_3_ powders	Reactive plasma spraying	Increased hardness and toughness	[164]
8	Mg, Mg_3_(PO_4_)_2_/AZ31 magnesium alloy	ZnO nanoparticles	Plasma electrolytic oxidation	Improved corrosion resistance	[55]
9	Trimethoxy(propyl)silane/steel	TiO_2_ nanoparticles	One-step electrophoretic deposition	Superhydrophobicity, decreased corrosion rate	[60]
10	Sintered oxides/Al	Al_2_O_3,_ SiO_2_, Al_2_(SiO_3_)_3_	Plasma electrolytic oxidation	Better corrosion resistance	[57]
11	Porous Ti scaffold	HA/TiO_2_	Sponge replication process and micro-arc oxidation	Improved osteoblastic activity of the porous Ti	[156]
12	Si_3_N_4_	TiN	Spark plasma sintering	High strength and high toughness; good erosion and corrosion resistance	[52]
13	HA/stainless steel	rGO-HA Ti-rGO-HA	Sol–gel technique	Bioactivity	[165]
14	Yttria-stabilized zirconia (YSZ) and aluminium oxide (Al_2_O_3_)	MWCNT	Vacuum hot-pressing technique	High stability, increased porosity	[166]
15	Al_2_O_3_	Graphene platelets	Metallurgical route	Resistance at elevated temperatures under critical load	[167]

## Data Availability

No new data were created.
